# Linking a Deep Learning Model for Concussion Classification with Reorganization of Large-Scale Brain Networks in Female Youth

**DOI:** 10.3390/bioengineering12090986

**Published:** 2025-09-17

**Authors:** Julianne McLeod, Karun Thanjavur, Sahar Sattari, Arif Babul, D. T. Hristopulos, Naznin Virji-Babul

**Affiliations:** 1 Department of Rehabilitation Sciences, University of British Columbia, Vancouver, BC V6T 1Z3, Canada; jdmcleod@student.ubc.ca; 2 Department of Physics and Astronomy, University of Victoria, Victoria, BC V8P 5C2, Canada; karun@uvic.ca (K.T.); babul@uvic.ca (A.B.); 3 School of Biomedical Engineering, University of British Columbia, Vancouver, BC V6T 1Z3, Canada; sattari.sahar@gmail.com; 4 School of Electrical and Computer Engineering, Technical University of Crete, 73 100 Chania, Greece; dchristopoulos@ece.tuc.gr; 5 Department of Physical Therapy, University of British Columbia, Vancouver, BC V6T 1Z3, Canada; 6 Djavaad Mowafaghian Centre for Brain Health, University of British Columbia, Vancouver, BC V6T 1Z3, Canada

**Keywords:** concussion, deep learning, classification, causal connectivity, information flow, EEG

## Abstract

Concussion, or mild traumatic brain injury, is a significant public health challenge, with females experiencing high rates and prolonged symptoms. Reliable and objective tools for early diagnosis are critically needed, particularly in pediatric populations, where subjective symptom reporting can be inconsistent and neurodevelopmental factors may influence presentation. Five minutes of resting-state (RS) EEG data were collected from non-concussed and concussed females between 15 and 24 years of age. We first applied a deep learning approach to classify concussion directly from raw, RS electroencephalography (EEG) data. A long short-term memory (LSTM) recurrent neural network trained on the raw data achieved 84.2% accuracy and an ensemble median area under the receiver operating characteristic curve (AUC) of 0.904. To complement these results, we examined causal connectivity at the source level using information flow rate to explore potential network-level changes associated with concussion. Effective connectivity in the non-concussed cohort was characterized by a symmetric pattern along the central–parietal midline; in contrast, the concussed group showed a more posterior and left-lateralized pattern. These spatial distribution changes were accompanied by significantly higher connection magnitudes in the concussed group (*p* < 0.001). While these connectivity changes may not directly drive classification, they provide evidence of large-scale brain reorganization following concussion. Together, our results suggest that deep learning models can detect concussion with high accuracy, while connectivity analyses may offer complementary mechanistic insights. Future work with larger datasets is necessary to refine the model specificity, explore subgroup differences related to hormone cycle changes and symptoms, and incorporate data across different sports.

## 1. Introduction

Concussion or mild traumatic brain injury (mTBI) remains a significant and growing public health concern globally. Concussions induce diffuse, heterogeneous, and spatially distributed changes in brain structure and function, resulting in cognitive, motor, emotional, and behavioral impairments that can persist for many months [1,2,3]. The annual rate of reported mTBIs is 1100 per 100,000 people [4]. Among youth, the burden is especially pronounced. A recent meta-analysis reported a pooled incidence of sports-related concussions (SRC) in children and adolescents (<18 years of age) of 1.41 per 1000 athletes exposure, and 4.36 per 1000 player hours across seven contact sports [5].

Importantly, female athletes experience a higher risk of concussion in contact sports, and report more severe symptoms and greater deficits in neurocognitive performance in comparison with males [6]; however, they remain significantly underrepresented in concussion research [7]. Reflecting broader trends in neuroscience research [8], the concussion literature has historically predominantly focused on male athletes, creating gaps in our understanding of sex-specific differences in injury mechanisms, symptom presentation, and recovery trajectories. This discrepancy underscores the critical need for *sex-specific* investigations to improve diagnostic accuracy, therapeutic interventions, and ultimately clinical outcomes for females affected by concussion.

Current diagnostic approaches rely heavily on self-reported subjective symptom reports and clinical assessments, which are prone to variability and may not accurately reflect underlying neural dysfunction [9,10]. Neuroimaging and electrophysiological techniques have been employed in research for several decades to enhance the understanding of concussion pathophysiology and work toward more objective diagnoses. Electroencephalography (EEG), in particular, offers a non-invasive, cost-effective method for assessing brain function by capturing real-time electrical activity. Traditional EEG analyses, however, are limited by their reliance on manually extracted features, and may lack sensitivity of the complex and dynamic nature of brain activity affected by concussion. Advances in EEG technology, coupled with increasingly sophisticated and analytic methods, have enabled researchers to detect brain alterations post-injury [11,12,13,14,15,16].

Numerous studies have shown that the brain is highly active during the resting state, with a highly sophisticated temporal connectivity structure that is characterized by spontaneous fluctuations across spatially distributed regions. In addition, the resting state of the brain utilizes approximately 80% of the brain’s energy in supporting a number of functional tasks such as perception, working memory, etc., while task-related metabolic changes account for less than 5% [17]. This intricate interplay achieves a balance between efficient information processing and metabolic expenditure, which maintains the brain in a highly responsive state [18] and is very sensitive to changes in the brain that can be picked up by EEG.

Recent advances in artificial intelligence, particularly deep learning algorithms, have revolutionized EEG analysis by enabling automated, data-driven classification of neurological states. Deep learning models, such as long short-term memory (LSTM) recurrent neural networks, can automatically learn and detect temporal patterns in *raw* EEG data, without the need for manual feature selection. We have previously shown high accuracy in classifying concussions (i.e., [19,20]). In addition, Daly et al. [21] have successfully applied this approach for neonatal seizure detection, showing some generalizability of this approach. Despite their predictive success, deep learning models tend to function as “black boxes”, offering limited insight into the reasoning behind their classifications, and thereby constraining the related interpretability about the neural mechanisms that underlie the EEG changes. This limitation presents a challenge for clinical translation, where insights about the mechanistic disruptions in brain function following injury are essential for developing targeted interventions and objective injury assessment.

Brain connectivity metrics, particularly causal connectivity derived from EEG source data, offers insights into the directional flow of information between brain regions, and can be used to reveal disruptions in network-level neural communication following concussion. We were the first to show changes in effective connectivity as measured by EEG using information flow rate [22]. Our analysis shows that the dominant nexus of information flow in healthy male adolescents was primarily left-lateralized and anterior-centric, characterized by strong bidirectional information exchange between the frontal regions, and between the frontal and the central/temporal regions. In contrast, adolescent males with concussion showed distinct differences in information flow marked by a more left–-right symmetrical, albeit still primarily anterior-centric, pattern of connections, diminished activity along the central–parietal midline axis, and the emergence of inter-hemispheric connections between the left and right frontal and the left and right temporal regions of the brain. We also found that the statistical distribution of the normalized information flow rates in each group (non-concussed and concussed) was significantly different. This was a critical first step towards understanding information flow in the brain during a key transition stage between childhood and adulthood and the impact of brain injury on effective connectivity [22].

Recently, Reddy et al. [23] found increased posterior-to-anterior effective connectivity following concussion in a sample of male high-school football players. In a pediatric case–control sample, Vaughn et al. [24] found that post-concussion effective connectivity within the DMN showed increased inter- and intra-hemispheric anterior connectivity, with unique connections from the orbitofrontal cortex to parietal regions. In adults, acute concussion has been associated with decreased connectivity from the left middle frontal gyrus to various areas in the temporal, frontal, and insular regions, from the left insula to areas within the frontal and central regions, and from the right insula to the left superior frontal gyrus. Increased connectivity was observed from the left anterior cingulate cortex to areas of the frontal lobe and the left insula, as well as from the right insula to the left superior temporal gyrus [25,26].

These network-level disturbances suggest that concussion affects not only local neuronal activity, but also the broader organization of neural information flow. Integrating this approach with deep learning-based EEG classification could reveal whether micro-level EEG disruptions predict broader connectivity changes, providing a promising avenue for elucidating the neural underpinnings of concussion.

The main goal of this research is twofold. First, to evaluate the performance of our previously developed, and tested in males, deep learning neural network-based classifier—hereafter referred to as the Concussion Classification Network (ConcNet)—in assessing the accuracy of concussion classification in females. Second, to apply causal brain connectivity analyses to characterize differences in network organization between groups. By combining automated classification with network neuroscience, this study aims to bridge the gap between data-driven concussion detection and mechanistic understanding to provide insights relevant to advancing diagnostic accuracy, recovery, and the clinical management of concussion.

## 2. Materials and Methods

### 2.1. Participants

Female athletes between 15 and 24 years of age were recruited for this study. Participants were recruited from various contact and non-contact sports teams, including soccer, rugby, and swimming. The inclusion criteria for non-concussed athletes was no history of concussion; concussed participants were required to be within 30 days of injury, with a diagnosis of concussion from a physician or team doctor, symptomatic at time of study enrolment (reported > 4 symptoms on the SCAT5, in line with normative reference values for female rugby players [27]), and have reported no medical clearance to return to play (RTP). Exclusion criteria for all participants included focal neurologic deficits and/or diagnosis with a neurological condition.

This study was approved by the University of British Columbia Clinical Research Board (H17-02973), in accordance with the Declaration of Helsinki. All participants provided written informed consent before participating.

### 2.2. Concussion Symptom Assessment

The Sport Concussion Assessment Tool 5 (SCAT5) was used for assessing the number and severity of concussion symptoms. The SCAT5 is a standardized sport concussion assessment tool that is used to evaluate the presence and severity of concussion symptoms [28]. Respondents were asked to rate the severity of a series of 22 symptoms on a 6-point scale ranging from 0 (none) to 6 (severe). Two scores are generated from the questionnaire: total number of symptoms and total severity score. The SCAT5 data were collected at the time of EEG recordings.

### 2.3. EEG Acquisition

Five minutes of resting-state eyes-closed EEG data were collected for all participants using a 64-channel HydroCel Geodesic Sensor Net (EGI, Eugene, OR, USA) connected to a Net Amps 400 high-impedance amplifier. The vertex (Cz) served as the reference point during the recording setup. Data were recorded at a 500 Hz sampling rate, with scalp electrode impedances typically less than 50 kΩ.

### 2.4. Data Preprocessing for Deep Learning

For the deep learning approach, our methodology relies on using *raw* EEG data with minimal preprocessing as the input to the neural network. In the reported deep learning literature, methods for the preprocessing of EEG data, such as bandpass filtering and artifact removal, are commonly used. However, there is no widely accepted, standard preprocessing methodology in the literature. Ad hoc approaches may lead to subjective bias and difficulty in achieving reproducible results; therefore, our objective is to use minimal preprocessing to avoid any bias introduced in these steps, as per our previous work [19,20].

Our deep learning approach was built using Python v3.10 and Tensorflow v2.12 framework. EEG recordings were first converted from the proprietary EGI format to Python Numpy data format. Four seconds were trimmed at the start and the end of each 5 min long recording to remove any transient noise. Five of the datasets had been recorded at a sampling rate of 250 Hz, while the remaining were recorded at 500 Hz. All the datasets were resampled to a uniform sample rate of 250 Hz. During data recording, the Net Amps amplifier applies a built-in hardware low-pass filter, with the upper frequency automatically set to half the sampling rate to ensure Nyquist sampling. Thus, data recorded with sample rate of 250 Hz had been low-pass filtered at 125 Hz during data recording, while datasets recorded at a 500 Hz sampling rate had been filtered at 250 Hz. To make the dataset uniform, all datasets were low pass filtered at 100 Hz using a digital 5th order Butterworth filter.

EEG signals are non-stationary; therefore, smaller segments of a recording can be treated as independent data as a way of augmenting limited datasets. Adopting this approach, the 5 min long EEG recording from each participant was segmented into contiguous epochs of 10 s length for use in training and testing the networks. This led to a nominal 30-fold increase in the size of the dataset. In order to prevent data leakage between the training and testing datasets, all segments from any given participant were included only in the training dataset or in test dataset, and not split between the two.

### 2.5. Deep Neural Network Architecture

**Figure 1:**
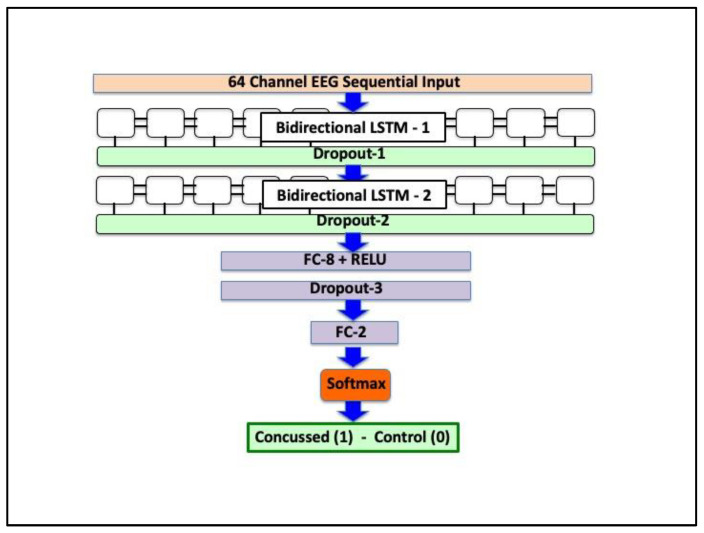
Schematic diagram showing the architecture of ConcNet, the recurrent neural network used for concussion classification.

ConcNet [19,20] is based on a recurrent neural network (RNN) architecture, which employs LSTM units to process and extract latent features from the multi-channel EEG recordings. These extracted latent features form the input to fully connected layers used for classification. The ConcNet architecture is schematically shown in Figure 1. The 64-channel EEG data is input through a sequential input layer into a bilinear LSTM layer comprising 24 recurrent units. The output from the bilinear LSTM layer is regularized through dropout with probability 0.32 and input to a second bilinear LSTM layer with 24 units, followed by dropout with probability 0.32. The extracted latent features are input to two fully connected layers for classification. The first fully connected layer has 8 hidden units and RELU activation regularized by dropout with probability 0.32. The final fully connected layer with two units outputs to a softmax layer, which assigns probabilities, PConc and PHlth (=1 − PConc) for the input EEG data to be concussed or non-concussed based on PConc being greater than a user-chosen threshold of 0.5.

For regularization, we used dropout (DO) with a probability of 0.32 after each bLSTM layer and after the first fully connected layer. We used cross-entropy loss function as the classification criterion, and an ADAM optimizer with a learning rate of 0.03. Standard mini-batch gradient descent was used with a batch size of 12 and 5 epochs for training.

ConcNet was implemented using Tensorflow version 2.11 with Keras backend. Training and testing of the networks were carried out on Digital Research Alliance of Canada clusters with one GPU assist. Hyperparameters were tuned using Bayesian optimization within the Keras Tuner framework.

A custom K-fold cross validation scheme was used for training and test datasets in a user-chosen ratio, 80% to 20% in our case. Since the EEG dataset contains several segments from the same participant, the dataset was split based on all the participants to ensure that all segments from any given participant were included in either the training or the test set, and not in both, thus avoiding any data leakage between them. In addition, we ensured that the training dataset contained the same number of concussed and non-concussed participants to maintain an even balance between the two classes.

Standard performance metrics, namely, accuracy, recall, precision, and specificity, which are commonly used to assess network performance in binary classification tasks, were calculated. Furthermore, the performance of ConcNet was based on two standard metrics: the confusion matrix (CM), and the area under the curve (AUC) of the receiver operating characteristic (ROC) curve. The CM is a visual representation of the performance metrics of a network used for classification. For binary classification, the four cells in the CM plot show the true positive and negative rates, as well as the false positives and negatives. From these four values, we computed all the standard performance metrics of the network. The CM and the corresponding 95% CI values were computed using the ground truth and predicted labels of the test samples in the five repeats of the 6-fold cross validation tests. The ROC traces the true positive rate (TPR) against the false positive rate (FPR) as a function of the classification threshold. An ideal network would have a TPR of 1, and FPR of 0 for all classification thresholds. AUC, the area under the ROC, represents the probability that the network will output a higher prediction score when presented a test sample drawn at random from the concussed class than from the non-concussed class. A classifier with no better accuracy than chance would be expected to have an AUC of 0.5, while an AUC of 1 corresponds to a classifier with perfect accuracy.

### 2.6. EEG Preprocessing and Source Reconstruction for Causal Connectivity Analysis

**Figure 2:**
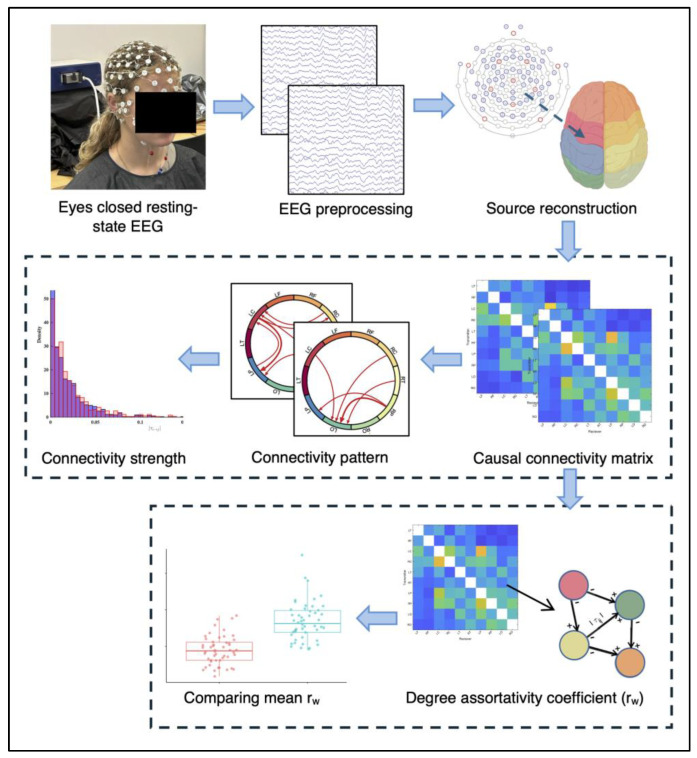
Study design and analysis procedure for causal connectivity and degree assortativity analysis.

Raw EEG data were preprocessed using EEGLAB in MATLAB R2022b [29]. Data were re-referenced to the average of all channels, downsampled to 250 Hz, notch-filtered, and bandpass-filtered between 0.5 and 50 Hz. Non-brain artifacts (i.e., noise caused by participant motion) identified through independent component analysis (ICA) and visual inspection were removed. Figure 2 shows the procedure of EEG data collection and analysis for the causal connectivity analysis.

Clean EEG sensor-level data were transformed to source space using Brainstorm in MATLAB [29]. An MRI head template (ICBM152) was used for head modeling [30]. The head model was divided into three sections (scalp, skull, brain) for forward modeling. The source space was constrained to the cortex. Minimum norm estimate (MNE) and sLORETA were used for inverse modeling [31,32]. The cortical surface was symmetrically divided into ten regions of interest (ROIs) using the Desikan–Killiany atlas [33].

The Desikan–Killiany atlas was selected because it is commonly used for surface EEG analyses, and because it provides a coarse sampling of the cortex. This was ideal for our research question as it allowed us to focus on broad cortical patterns. The ten predetermined ROIs were as follows: left-frontal (LF), right-frontal (RF), left-central (LC), right-central (RC), left-parietal (LP), right-parietal (RP), left-temporal (LT), right-temporal (RT), left-occipital (LO), and right-occipital (RO). Each of the ten source regions generated a distinct time series, assumed to reflect the sum of electrical activity occurring in that specific brain area. The resulting clean source reconstructed EEG data were used to calculate causal connectivity.

### 2.7. Causal Connectivity

Causal connectivity was quantified using Liang–Kleeman’s information flow rate (IFR) [22,34]. IFR provides a framework for measuring the transfer of information in dynamic systems and as previously mentioned, it is particularly suitable for time series data since it does not require the assumption of stationarity [35]. The theorem is briefly described in the following section; for comprehensive review of the framework, see [34].

The covariance between two source regions, $v_{i}$ (transmitting information) and $v_{j}$ (receiving information), is computed with the sample cross-covariance formula. This calculation quantifies the extent to which time series $v_{i}$ and $v_{j}$ are correlated over time. Specifically, it assesses how fluctuations in $v_{i}$ are associated with the fluctuations in $v_{j}$, examining deviations from their average values over time. For dipoles located at $v_{i}$ and $v_{j}$, the sample cross-covariance coefficient is denoted as ${\hat{C}}_{i,j}.$ The magnitude (strength of electrical activity) at source location $v_{i}$ and at time point *n* is $v_{i,n}$; for source location $v_{j}$, $v_{j,n}.$ The computation of the sample cross-covariance uses the following formula:

(1) $${\hat{C}}_{i,j}=\bar{v_{i,n}v_{j,n}}-\bar{v_{i,n}}\;\bar{{\;v}_{j,n}},\;\mathrm{for}\;i,\;j=1,\ldots .,10$$

where $\bar{v_{i,n}}$ and $\bar{{\;v}_{j,n}}$ represent the mean values of $v_{i,n}$ and $v_{j,n}$, respectively.

The Pearson correlation between time series $v_{i}$ and $v_{j}$ $({\hat{r}}_{i,j}$) is a normalized measure of the linear relationship between the activities of two brain regions. Both ${\hat{C}}_{i,j}$ and ${\hat{r}}_{i,j}$ are non-directional and ${\hat{r}}_{i,j}$ is commonly reported as a measure of functional connectivity. The *cross-correlation coefficient* between $v_{i}$ and $\dot{v_{j}}$ (the temporal derivative of $v_{j}$) over time and is denoted as ${\hat{r}}_{i,dj}$. This quantifies how the activity in one brain region relates to the rate of change in the activity of another region. The formulas used to estimate ${\hat{r}}_{i,j}$ and ${\hat{r}}_{i,dj}$ are

(2) $${\hat{r}}_{i,j}=\frac{{\hat{C}}_{i,j}}{\hat{\sigma_{i}}\hat{\sigma_{j}}},\;\mathrm{for}\;i,\;j=1,\ldots .,10$$

(3) $${\hat{r}}_{i,dj}=\frac{{\hat{C}}_{i,dj}}{\hat{\sigma_{i}}\hat{\sigma_{j}}},\;\mathrm{for}\;i,\;j=1,\ldots .,10$$

where $\hat{\sigma_{i}}=\sqrt{{\hat{C}}_{i,j}}$ is the sample standard deviation of $v_{i}$ and ${\hat{C}}_{i,dj}$ is the sample covariance of $v_{i}$ and first derivative of $v_{j}.$

The information flow rate from the time series $v_{i}$ to $v_{j}$ ($T_{i\to j}$) is calculated using the Liang–Kleeman coefficient formula. To evaluate the significance of the information flow, the normalized information flow rate (*τ_i→j_*) is calculated. The coefficient *τ_i→j_* measures the proportion of total entropy rate change in ${\;v}_{j}$ attributed to $v_{i}$. Computation of *τ_i→j_* uses a normalization factor ($Z_{i\to j}$) that is further described in [34]. Higher |*τ_i→j_*| values signify greater information transfer between regions. A threshold of 0.05 has been used to identify active connections, indicative of meaningful influence between brain regions.

(4) $$T_{i\to j}=\frac{{\hat{r}}_{i,j}}{1-{{\hat{r}}_{i,j}}^{2}}\left({\hat{r}}_{i,dj}-{\hat{r}}_{i,j}{\hat{r}}_{j,dj}\right),\;\mathrm{for}\;i,\;j=1,\ldots .,10,\;i\neq j$$

(5) $$\tau_{i\to j}=\frac{T_{i\to j}}{Z_{i\to j}}$$

The resulting output from the casual connectivity computation for a single participant is a binary weighted directed network matrix with the magnitude (strength) of each connection corresponding to |*τ_i→j_*|. Connectivity matrices for individuals were averaged to produce a single matrix for concussed and non-concussed groups, followed by a between-group comparison.

### 2.8. Degree Assortativity

As a secondary outcome measure of brain connectivity, we compared the degree of assortativity between the concussed and non-concussed groups. Assortativity is a summary statistic of a network’s topological structure. It was originally conceptualized by Newman et al. [36] and later studied in the context of brain connectivity [22]. It is a graph theory metric that calculates the tendency of nodes in a network to connect with other nodes of similar degree. Assortativity is expressed on a scale ranging from −1 (perfectly disassortative) to 1 (perfectly assortative) [37].

The degree assortativity coefficient (r_w_) was calculated using an adaptation of Newman’s original formula for weighted, directed networks, as described by Rubinov and Sporns [38]. The r_w_ is the Pearson correlation between the degree of nodes at either end of an edge [37]. The 10 predefined ROI source regions are the nodes, and the |*τ_i→j_*| are the weighted edges. The binary matrix generated from the casual connectivity calculation is used to determine the weight of each node. Each node is assigned an in-degree and an out-degree. The in-degree is calculated from the sum of the |*τ_i→j_*| for all incoming connections. The out-degree is calculated from the sum of the |*τ_i→j_*| for all outgoing connections. The pearson.W.m function from the Octave networks toolbox in MatLab was used to calculate r_w_ [39].

### 2.9. Statistical Analyses

A post hoc power analysis using parameters from [22] indicated that a sample size of *n* = 44 controls and *n* = 29 concussed would be required to detect a mean difference in connectivity strength with 80% power (effect size = 0.6823, α = 0.05). Descriptive statistics were computed for the demographic data and SCAT5 scores. Group differences in age and sport distribution were examined to assess comparability between concussed and control participants. Age was compared using Welch’s two-sample *t*-test and Mann–Whitney U tests. Level and type of sport was compared between groups using Fisher’s exact test. All tests were conducted at 5% significance. The analyses were completed using SPSS software (version 29.0.20.0).

The statistical significance of all mean connections was assessed. Using non-parametric permutation testing, we tested the null hypothesis that there was no information flow between $v_{i}$ and $v_{j}$. For each participant, the transmitter time series was permuted 100 times to generate 100 permuted |*τ_i→j_*| values for each connection. The significance threshold was set by calculating the 5th and 95th percentiles of these permuted distributions derived from the permutations.

The magnitudes of |*τ_i→j_*| were compared between both groups. One distribution per group was generated using |*τ_i→j_*| values for each of the 90 connection pairs between the 10 ROIs per individual in each group. Hence, the distributions consisted of 15 × 90 = 1350 |*τ_i→j_*| values in the non-concussed group and 11 × 90 = 1080 |*τ_i→j_*| values in the concussed group. Differences in the shape and central tendencies between the two distributions were assessed using the Kolmogorov–Smirnov and Kruskal–Wallis tests. In addition, 95% confidence intervals were reported for the coefficient of variation (COV), skewness, and kurtosis, corresponding to further described differences between the distributions. Descriptive statistics (mean, median, standard deviation, coefficient of variation, kurtosis, and skewness) were calculated for each group, and Cohen’s *d* was computed as an estimate of effect size. One hundred thousand (100,000) subsamples of six individuals were generated from random subsampling of each group, and plots were generated to visualize the resulting COV, skewness, and kurtosis of each distribution.

The r_w_ was calculated for each subject based on their individual matrix. Mean r_w_ was calculated by averaging all individual r_w_’s. Between-group comparison of mean r_w_ was conducted using an independent samples *t*-test. The independent samples effect size using Cohen’s d was reported.

## 3. Results

### 3.1. Clinical and Demographic Data

Table 1 presents the descriptive statistics for the concussed and non-concussed groups. Age for the entire sample (N = 26) ranged from 15 to 24 years: *n* = 11 with subacute concussion (M = 20; SD = 2.46) and *n* = 15 non-concussed participants (M = 21 years; SD = 1.88). Concussed participants were primarily involved in soccer and rugby, with one participant from ringette, whereas control participants were more evenly distributed across rugby, swimming, rowing, track, and ultimate frisbee. There was no statistically significant age difference between the concussed and control groups (*t*(18) = −1.11, *p* = 0.28). This finding was confirmed using a Mann–Whitney U test (W = 62.5, *p* = 0.30). The majority of participants played sports at the university level; Fisher’s exact test indicated no significant difference between groups (*p* = 0.35). All concussed participants reported a diagnosis of concussion and averaged 10 days post-injury (SD = 6.85). On average, the concussed group reported 14.88 (SD = 5.48) symptoms with a severity of 34.88 (SD = 22.53; range = 7–71) on the SCAT5.

### 3.2. ConcNet

The final dataset consisted of 15 non-concussed and 11 concussed participants. With the 80-to-20% split, the training dataset consisted of all the segments from eight non-concussed and eight concussed participants, while the test dataset had six non-concussed and two concussed participants. Segments from one concussed and one non-concussed participant were used as the validation dataset during training.

Since there were two concussed participants in each test dataset, a 6-fold cross validation approach was adopted to test all the concussed and non-concussed participants at least once. This was repeated five times, shuffling the participants at the start of each sequence so that each participant was classified by a network, which had been trained with different groups of participants, thus mimicking an ensemble learning approach.

For each repeat run of the 6-fold cross validation, the concussed participants and the non-concussed participants were shuffled, so that the resulting training and test datasets for the cross-validation were unique. Therefore, the performance of the network was assessed with 30 independent realizations of the training and testing datasets.

Table 2 lists the mean value and the lower and upper 95% confidence intervals of the performance metrics of ConcNet. The mean and corresponding 95% confidence intervals of the metrics were computed from the 30 independent realizations of the training and testing datasets in the five repeats of the 6-fold cross-validation tests.

ConcNet achieved an overall accuracy of 84.2% (81.0/87.3), indicating that, on average, the network correctly classified eight or more test samples, irrespective of the sample being drawn from the concussed or non-concussed class.

**Figure 3:**
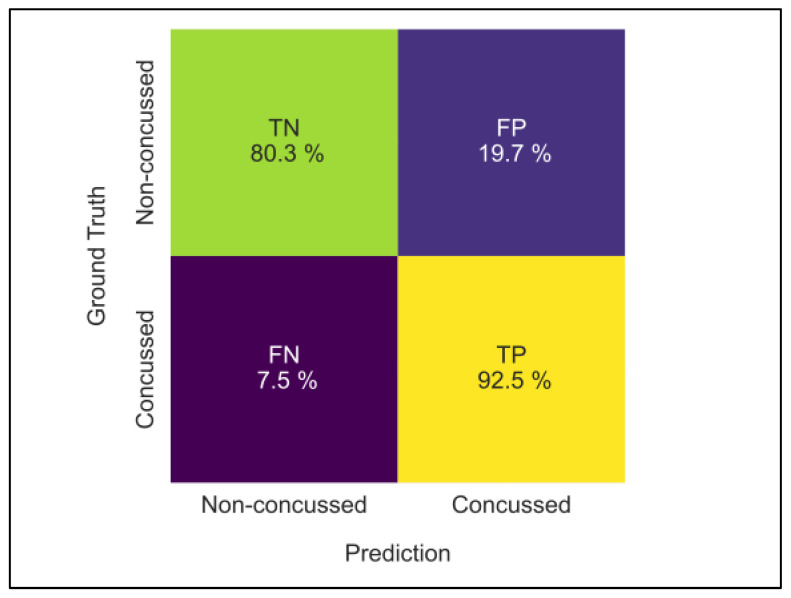
Confusion matrix showing the mean values of the true and false positive and negative values obtained from test samples used in the five repeats of the 6-fold cross validations.

For test samples drawn only from the concussed class, the recall, also termed the true positive rate (TPR) was higher at 92.9% (90.1/95.6), indicating that concussed participants are correctly classified more than nine times out of ten. Using only test samples drawn from the non-concussed class, the specificity, or true negative rate (TNR), was 75.7% (70/81.4). Therefore, on average, only three out of every four non-concussed participants were correctly classified, with the remaining non-concussed participant being misclassified as concussed, resulting in a false positive. This lower specificity of ConcNet leads correspondingly to a precision or the positive predictive value (PPR) of 92.5% (77.1/84.7). On average, out of any ten test samples classified by ConcNet as being concussed, only eight were truly concussed (true positives), with the other two being misclassified non-concussed participants (false positives). Figure 3 shows the mean values annotated on the CM.

**Figure 4:**
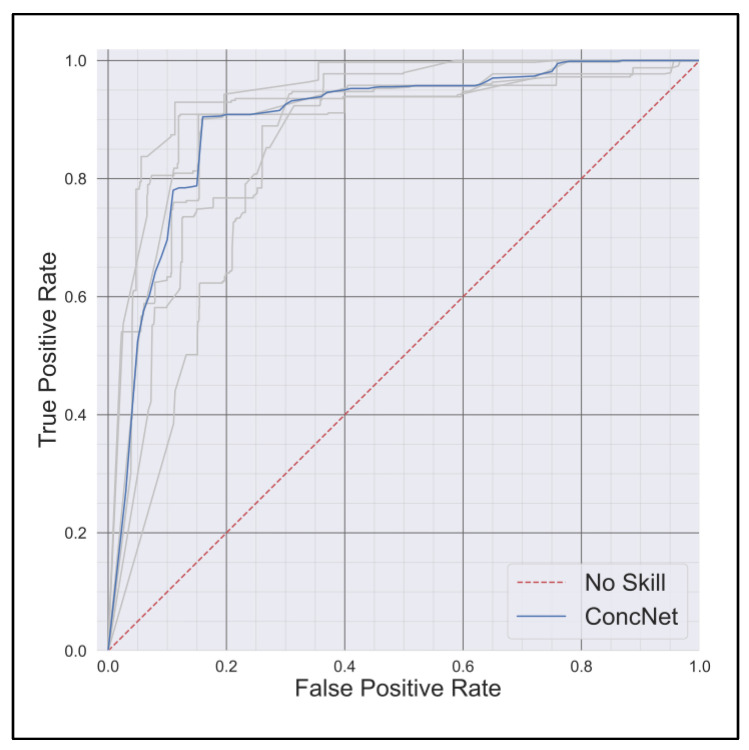
ROC curves for each of the five repeats of the 6-fold cross validation tests (in gray) and the corresponding median ROC curve (in blue). The diagonal 1:1 line shows the ROC of a network with no skill.

Figure 4 shows the receiver operating characteristic (ROC) curve of ConcNet derived from the five repeats of the 6-fold cross validations. The median ROC is estimated from the five trials. The mean and 95% CI of the AUC values were 0.904 (0.87/0.92). ConcNet demonstrated high sensitivity in detecting concussion with most concussed participants correctly classified and median probability values above the decision threshold; however, specificity was lower (75.5%), as several non-concussed participants were frequently misclassified. Probability distributions confirmed that while concussed cases were reliably identified, non-concussed cases showed greater variability, leading to reduced true negative rates.

**Figure A1:**
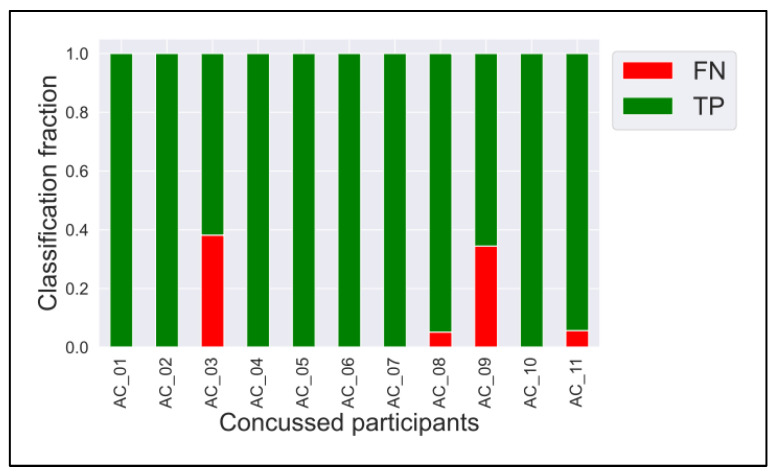
Fractional classification for each concussed participant. Green bars indicate correct classification; red bars indicate misclassification. In the concussed group, seven participants were always correctly classified, while two were misclassified less than 10% of the time. Participant AC_03 was misclassified 38.2% of the time. Participant AC_09 was misclassified 34.5% of the time.

**Figure A2:**
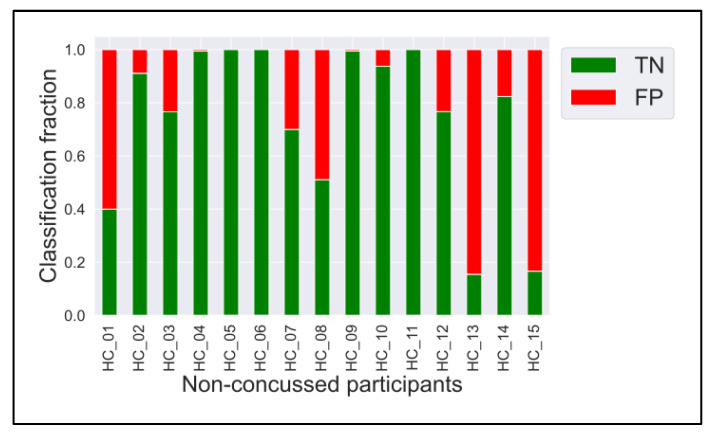
Fractional classification for each non-concussed participant. Green bars indicate correct classification; red bars indicate misclassification. Three participants (HC_01, HC_13, HC_15) were misclassified 60%, 84.5%, and 83.3% of the time, respectively. Further analysis showed that these three participants were rugby players. Please see the Discussion about the effects of subconcussive brain injury in rugby. Seven other participants were misclassified in the range of 6–49% of the time, while five were correctly classified 100% of the time.

**Figure A3:**
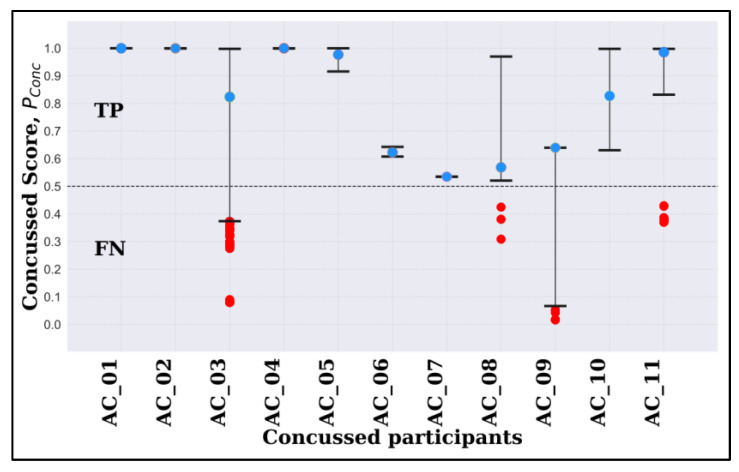
Distribution of the prediction probabilities output by ConcNet for concussed participants. P_conc_ (range is 0–1). Probability greater than 0.5 is classified as concussed. The median and inter-quartile distances of the distribution for all epochs for each participant during the 6-fold cross validation tests are shown. Blue dots indicate correct classification; red dots indicate misclassification. The median values of all participants are above the classification threshold with eight >= 0.8. Two participants (AC_03, AC_11) have epochs misclassified 38.2% and 5.7% of the time. The median values of the classification probability for all participants is consistently above the classification threshold.

**Figure A4:**
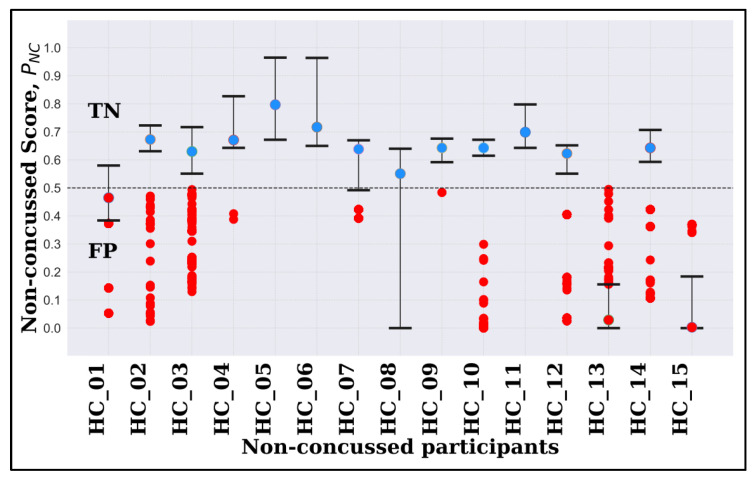
Distribution of the prediction probabilities output by ConcNet for concussed participants. P_NC_ (range = 1–P_conc_). Blue dots indicate correct classification; red dots indicate misclassification. The median values of 12 out of 15 participants are above the classification threshold but three are consistently misclassified (HC_01, HC_13, HC_15) with median values below the classification threshold.

Given the lower specificity relative to recall, we conducted additional analyses to examine the sources of misclassification and to determine whether errors reflected data quality issues or inter-individual variability. We examined misclassification patterns across individuals using repeated 6-fold cross-validation shown in Figure A1, Figure A2, Figure A3 and Figure A4, Appendix A. This analysis showed that errors were not randomly distributed. Specifically, three non-concussed participants (HC_01, HC_13, and HC_15) accounted for the majority of misclassifications (60–85% of the time), while five others were always correctly classified. Importantly, errors were not concentrated in specific noisy epochs but occurred across multiple segments, suggesting that misclassification reflects inter-participant variability rather than data quality issues. Further analysis revealed that the misclassified non-concussed participants were all rugby players. We discuss the implications of this in the Discussion.

We also analyzed the distribution of classification probabilities (Figure A4). For concussed participants, median probabilities consistently exceeded the decision threshold, supporting the high recall (92.9%). For non-concussed participants, most medians were also above threshold, though several were closer to the boundary, consistent with lower specificity.

### 3.3. Causal Connectivity

#### 3.3.1. Spatial Distribution

**Figure 5:**
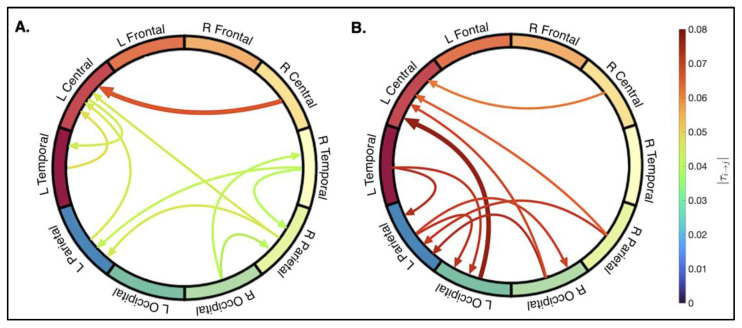
Spatial distribution of the strongest connections. The graph layout reflects the superior view of the cortical surface. Arrows represent the direction of causality, with arrow color corresponding to the magnitude (|*τ_i→j_*|) of the connection.

The spatial distributions of the ten strongest connections for the non-concussed and concussed groups are shown in Figure 5. The rank-ordered magnitudes and directions of these connections are listed in Table 3. For simplicity, only the ten strongest connections were qualitatively assessed, and show clear differences between the concussed and control groups.

In the non-concussed group, the top ten connections are widespread. The strongest connections are predominantly within anterior regions, with the top connection transmitted from the right- to left-central area. The left-central region is a primary hub for receiving information. Three inter-hemispheric connections are observed, including right-to-left communication between temporal and parietal areas. Left-sided intra-hemispheric connections are more focused in anterior regions (LC→LT, LT→LC, LP→LC), while right-sided intra-hemispheric connections are more localized the posterior region (RO→RT, RO→RP, RT→RP).

In the concussed group, connectivity is characterized by a posterior and left-lateralized pattern. The strongest connection is between the left-occipital to the left-central region. The left-occipital and -parietal areas are primary hubs for incoming and outgoing connections. There is an increase in strong inter-hemispheric connections (RO→LP, RO→LC, RP→LP, RP→LC, RC→LC) and a decrease in right intra-hemispheric information flow.

Common connections for both groups include RC→LC, RP→LC, RP→LP. Unique to the non-concussed group are connections transmitted from RT. Information transmitted from the right occipital is consistent between the two groups, but its targets differ: RO→RT and RO→RP in the non-concussed group, and RO→LC and RO→LP in the concussed group. The concussed group shows more information flow to and from the left-occipital (LO→LC, LT→LO, LP→LO) and left-parietal (incoming connections from the LT, RO, and RP and outgoing to RO and LO) regions. The left-temporal region transmits information posteriorly in the concussed group (LT→LO, LT→LP), compared to anteriorly in the non-concussed group (LT→LC). The left-central region is a main receiver of inter- and intra-hemispheric information in both groups, but only the non-concussed participants show a strong outgoing connection.

#### 3.3.2. Magnitude

**Figure 6:**
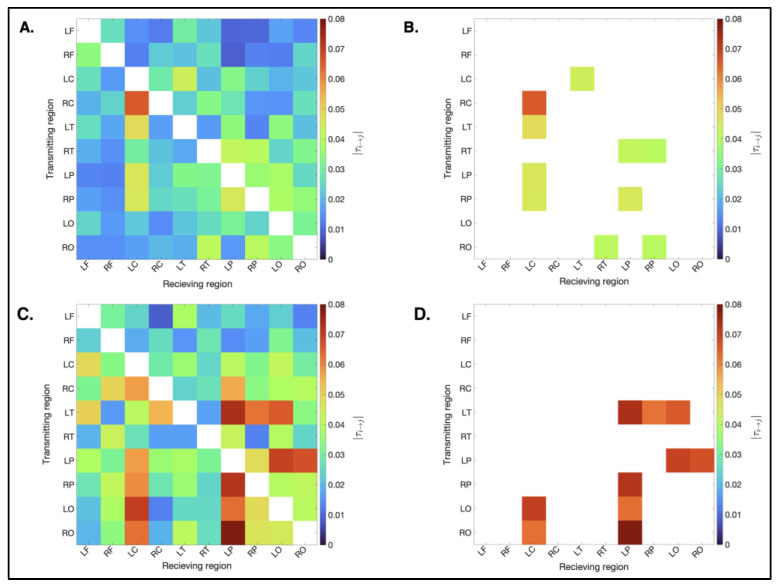
Causal connectivity matrices for the non-concussed and concussed groups. Panels (**A**,**C**) illustrate the mean |τ_i→j_| for each of the 90 possible pairs of the ten source ROIs for the non-concussed and concussed groups, respectively. Panels (**B**,**D**) illustrate the ten strongest connections for the non-concussed and concussed groups, respectively.

Figure 6 shows the binary, weighted matrices showing the mean absolute normalized information flow rates (|*τ_i→j_*|) for the concussed and non-concussed groups. Overall, |*τ_i→j_*| values were higher in the concussed group. The difference in magnitude of |*τ_i→j_*| is visible in the plots due to the color bar scale. The concussed group matrix shows higher magnitudes (red), which is particularly clear in Figure 6B,D, where only the ten most active connections are shown.

As listed in Table 3, the magnitudes of the top ten connections in the non-concussed group ranged from 4.01 × 10^−2^ to 6.54 × 10^−2^, with only one connection (RC→LC) exceeding the active threshold of 0.05. In contrast, the top ten connections in the concussed group ranged from 6.10 × 10^−2^ to 7.63 × 10^−2^, with 19 connections exceeding the active threshold. For the list of all 90 rank-ordered connections refer to Appendix B.

#### 3.3.3. Degree Assortativity

**Figure 7:**
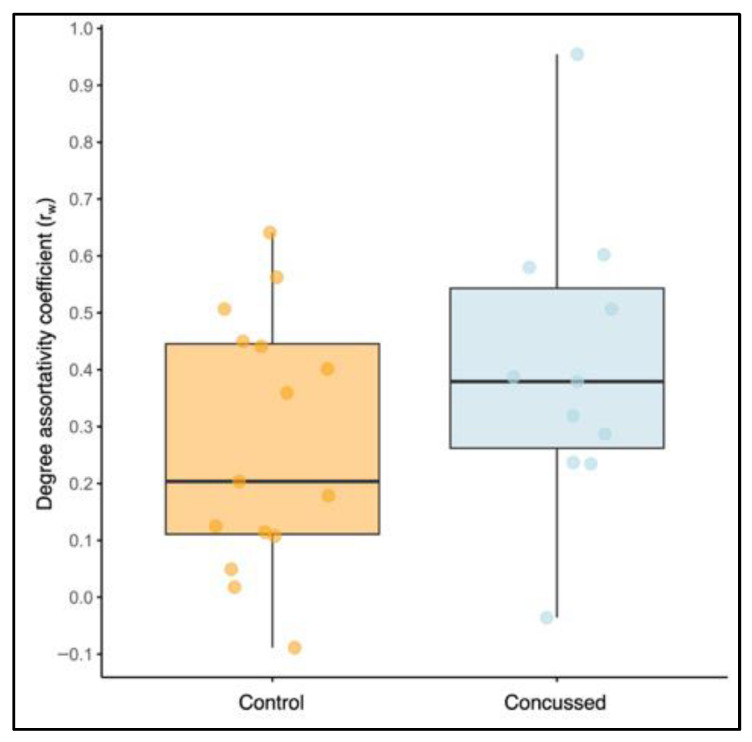
Boxplot comparing the degree assortativity coefficient (r_w_) between the non-concussed and concussed groups. Each dot represents an individual participant’s degree assortativity coefficient.

The mean r_w_ was higher in the concussed group (M = 0.41, SD = 0.26) compared to the non-concussed group (M = 0.27, SD = 0.22) (Figure 7). However, an independent samples *t*-test assuming unequal variances found no statistically significant difference between the groups, t(19.79) = 1.39, *p* = 0.18. The mean difference was 0.13 (SE = 0.09), with 95% CI equal to [−0.07, 0.33], with Cohen’s d indicating a small effect size (i.e., the difference in the means is small compared to the variability), *d* = 0.24, with 95% CI [−0.24–1.35].

#### 3.3.4. Statistical Analysis for Causal Connectivity

First, we determined the statistical significance of all 90 mean connections in each group. The 95th percentile of the |*τ_i→j_*| randomized distribution derived by means of permutation testing were 1.38 × 10^−5^ for the non-concussed group and 1.75 × 10^−5^ for the concussed group. Since all mean connections exceeded these thresholds, statistical significance was confirmed for both groups.

**Figure 8:**
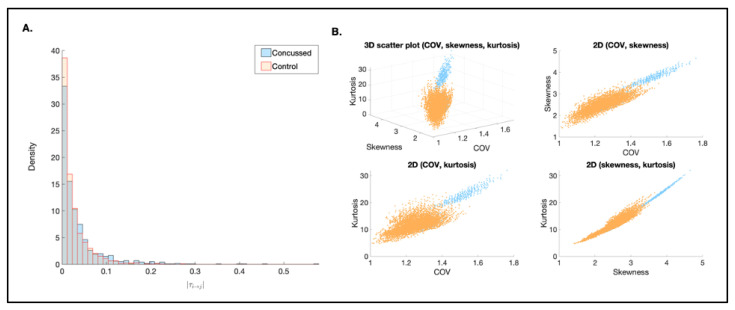
(**A**) Probability density histogram comparing two distributions (non-concussed and concussed). All individuals are included in each group. The density (y) of individual connections at each given magnitude (x) illustrates the overall strength of causal connections in each group. (**B**): scatter plots of COV, skewness, and kurtosis derived from 100,000 subsamples, each comprising six randomly selected individuals. The COV, skewness, and kurtosis are based on all the connections and individuals in each subsample. Orange points indicate the non-concussed group, and blue points indicate the concussed group.

Next, we generated a probability density histogram consisting of two distributions comprising all individuals in each group. The probability density histogram (Figure 8A) showed that the non-concussed group was more likely to have lower mean |*τ_i→j_*| values compared to the concussed group. The Kolmogorov–Smirnov (K-S) and Kruskal–Wallis (K-W) test indicated that the distributions are significantly different (*p* = 1.61 × 10^−5^ and *p* = 1.50 × 10^−3^, respectively). In other words, the |*τ_i→j_*| values were significantly higher in the concussed group in comparison to the control group.

Group-level descriptive statistics are summarized in Table 4. The mean information flow rate was slightly higher in the concussed group (0.0364) compared to the control group (0.0251), with similar median values. Both groups showed high coefficients of variation, reflecting considerable within-group variability. Distributional characteristics also differed, with the concussed group showing higher kurtosis and skewness, suggesting a more peaked and asymmetric distribution (Figure 8B). Despite these differences, the effect size was small (*d* = 0.0502), indicating minimal group differences in connectivity magnitude.

Lastly, we selected a subgroup of individuals from each group and derive subsampling distributions of information flow rates to further evaluate characteristics of each group’s distribution (Figure 8B).

Even though the distributions derived from subsampling follow the same orientation, the distributions are fairly well separated and the shapes generally differ between the two groups. Although the two groups are not completely separated in this three-dimensional feature space, the observed behavior is in overall agreement with the results from the K-S and K-W tests, which indicated that the distributions are distinct from one another.

## 4. Discussion

In this study, we present a novel approach to first classify concussion by applying a recurrent neural network (ConcNet) to raw, minimally processed RS-EEG, and subsequently, use source-based causal connectivity to provide mechanistic insights into the dynamics of large-scale brain re-organization following concussion in female youth. Our findings demonstrate that ConcNet accurately classified concussion with an average accuracy of 84.2%, a F1 score of 86.2% and a median AUC of 0.904. This classification performance is consistent with previous studies that have demonstrated the utility of deep learning models in EEG-based concussion classification [19,20,21]. Our work is unique in its focus on female athletes, who are underrepresented in the literature, despite having higher concussion rates and more severe symptomatology [40].

ConcNet demonstrated particularly high recall, indicating that concussed EEG signals carry distinct temporal–spectral patterns that the model consistently detected; however, we did note a drop in specificity—similar to that in our earlier work on concussion classification with ConcNet using adolescent male data [20]. We could simply ascribe these misclassifications to statistical fluctuations normally associated with any deep learning classification work. However, the misclassifications are higher in the non-concussed participants than in the concussed, indicating that the reasons may be more systematic in nature. In this binary classification context, systematic misclassification of non-concussed participants may be either due to the network not seeing a *consistent* pattern associated with the non-concussed class, to an anomalous pattern in misclassified participants which is not seen in the majority of the non-concussed participants, or identifying a pattern associated with the concussed class in these misclassified non-concussed participants.

While ConcNet achieved high recall, the lower specificity highlights an important limitation. Our exploratory analyses showed that misclassifications were largely attributed to a small subset of non-concussed participants, with three consistently misclassified across cross-validation runs. Notably, three of the four consistently misclassified non-concussed participants were rugby players. It is highly likely that although these players were asymptomatic and non-concussed at the time of testing, they may have been exposed to subconcussive brain impacts. There is increasing evidence that repetitive subconcussive brain impacts in sports such as soccer and rugby may result in subtle neural changes that can be detected via EEG [41].

We further noted that errors were not concentrated in noisy epochs but occurred across multiple segments, suggesting that misclassification reflects inter-individual variability potentially related to subclinical effects of repeated head impacts, rather than data quality issues. Improving specificity will therefore require larger and more diverse non-concussed cohorts, careful screening for subclinical symptoms, and the inclusion of potential covariates that may influence functional connectivity. Addressing these factors will be critical to enhance model reliability and reduce false positives in clinical settings.

The high recall of 92.9% may indicate that ConcNet is able to identify a strong and distinct pattern in the EEG associated with concussion, which dominates over any inter-participant variations. This suggests that while concussion produces strong, characteristic neural signatures, variability in the non-concussed group may obscure a consistent “non-concussed” pattern. It is possible that without a clear pattern to aid classification, inter-participant variations may dominate, leading to lower median classification probabilities and lower specificity.

Although the deep learning classification results demonstrate that raw resting-state EEG from concussed participants can be distinguished from that of non-concussed participants, these metrics alone do not provide insights about the underlying neurophysiological changes driving the classification. To address this, we examined causal connectivity patterns to explore potential network-level changes in information flow between brain regions associated with mTBI.

### 4.1. Causal Connectivity

Our results show that in non-concussed athletes, the information flow is symmetric and centrally distributed, particularly along the central–parietal midline. In contrast, concussed participants exhibited more posterior and left-lateralized information flow, with the left-occipital and -parietal regions serving as primary hubs. This suggests that concussion disrupts anterior-central network hubs and may induce compensatory reorganization in posterior regions. These findings align with previous work demonstrating altered effective connectivity following concussion, including increased posterior–anterior connectivity [23] and aberrant interactions within the default mode and salience networks [24].

Vaughn et al. [24] also observed increased causal connectivity from the left-orbitofrontal cortex to the right-parietal regions following concussion. Similarly, we observed an increase between the right-parietal and left-central region in our concussed sample, though in the opposite direction (RP→LC). The opposite directionality may be attributed to age differences, as Vaughn et al. [24] studied a younger sample. Consistent with this interpretation, Michels et al. [42] reported anterior-to-posterior information flow in younger participants and posterior-to-anterior information flow in older participants. Vaughn et al. [24] found that stronger orbitofrontal-to-parietal connectivity correlated with fewer post-concussion symptoms. In adults, increased frontal connectivity has similarly been linked to fewer symptoms [43]. Our study shows that a similar pattern of increased frontal–parietal connectivity exists in female youth following concussion.

Information flow changes may reflect injury to white matter tracts, particularly the fibers of the corpus callosum, resulting from impact. Given the observed shift from a balance of inter- and intra-hemispheric information flow to a predominant left-hemispheric pattern, it could be hypothesized that structural damage led to the rerouting of communication pathways [44].

Our observation of reductions in specific connections in the concussed cohort lends additional support to the hypothesis that the balance of inter- and intra-hemispheric connections shift following concussion, with some connections becoming stronger and others becoming weaker. The result of this may be reflected in alterations in cognitive control. Li et al. [25,26] reported reduced connectivity from the left prefrontal cortex to the left middle temporal gyrus, from the left insula to regions of the prefrontal cortex, and from the left insula to the right Rolandic operculum. They speculate that these reductions may be associated with reduced information processing, poor working memory, emotional dysregulation, and poor cognitive control.

The concussed sample had significantly more active connections in comparison to the non-concussed group. This is suggestive of hyperconnectivity, which would indicate that, even at rest, the concussed sample exerted more effort. This aligns with numerous reports of increased functional connectivity in the acute phase of concussion [16,43,45,46,47,48,49,50,51,52,53]. Hyperconnectivity in the acute phase of injury might be an adaptive response, potentially occurring to re-establish network communication or to allocate resources for repair [44,54,55] of microstructural lesions and/or neurometabolic alterations [56,57,58]. While potentially beneficial in the short term, chronic hyperconnectivity has a high metabolic cost that can lead to resource depletion and neurodegeneration [44]. Disruptions in functional connectivity have been observed in adult and pediatric populations with persistent symptoms [59,60,61,62,63,64], as well as recovered athletes that have been cleared for RTP [65,66,67,68,69]. Further research is needed to determine whether the increased information flow we observed represents a transient compensatory response or a marker of ongoing network disruption.

We have previously investigated how pediatric concussion alters the temporal dynamics of brain states within resting-state networks using resting-state fMRI data [70]. Functional networks in resting state are not stationary, but switch between different brain states. The strength and the direction of connections vary from seconds to minutes [71,72,73]. Using a sliding window analysis, we extracted three separate brain states within the resting-state condition in both healthy adolescents and adolescents with concussion. We found that healthy adolescents switched dynamically between three brain states, spending approximately the same time in each brain state. In contrast, adolescents with concussion spent the majority of time in only one brain state. We hypothesize that this lack of dynamic flexibility is likely to negatively impact the performance of tasks that require shifting of attentional states or performance of more complex tasks [74].

The concussed group had a slightly higher mean degree assortativity coefficient (r_w_) compared to the non-concussed group; however, this difference was not statistically significant. This suggests that the two groups did not differ in topological structure, and since the mean r_w_ exceeded 0 in both groups, the networks were considered assortative. The degree assortativity coefficient is reflective of network robustness and interconnectivity [75]. Highly assortative networks are less likely to become disconnected after trauma, whereas disassortative networks are more vulnerable [37]. Greater assortativity suggests resilience, and might be an adaptive response to injury [38]. Although Churchill et al. [76] reported decreased assortativity after concussion, their study included an equal distribution of university-aged males and females, but did not report female-specific assortativity scores. Our concussed sample did not show this adaptive response, which could be an indication that the female response to concussion may differ from what has been reported in the literature. To our knowledge, there are no other studies that have evaluated degree assortativity coefficient following concussion in females. Our results hint sex-specific responses to brain injury that need further study.

While ConcNet achieved strong predictive performance, the interpretability of deep learning models remains a challenge with limited insight into the neural underpinnings of classification decisions [77]. In this study, we also examined causal connectivity to explore potential alterations in large-scale brain networks following brain injury. Although these connectivity results are not directly linked to the classification results, they provide complementary evidence of network-level reorganization, offering potential mechanistic insights. Together, these parallel approaches highlight the value of combining predictive modeling with network neuroscience perspectives. Future work using explainable deep learning tools could help determine whether these connectivity patterns are directly leveraged by the model, further bridging the gap between predictive accuracy and mechanistic interpretation.

### 4.2. Benefits of EEG Classifiers over Other Neuroimaging Techniques

EEG classifier models based on raw EEG data have a number of important benefits over other methods. First, our novel approach of using raw resting-state EEG data removes the need for extensive preprocessing steps that are typically required in conventional EEG analysis pipelines, such as artifact rejection, feature engineering, or source localization. This streamlines the workflow, reduces operator dependency, and enhances reproducibility, paving the way for rapid, automated deployment in clinical and field settings. Second, EEG classifiers do not require a baseline measurement when generating a prediction. This makes an EEG classifier more practical, allowing it to be applied following impact without the need for comparison with baseline behavioral measures [78]. Third, EEG is affordable and portable compared with detection methods such as functional MRI.

In addition, EEG has superior temporal resolution, capturing millisecond-level brain dynamics that are critical for identifying transient or subtle disruptions associated with sub-concussive impacts [41]. EEG can also detect functional brain changes that often precede structural abnormalities visible on imaging, making it particularly sensitive for early intervention. The classifiers provide objective, quantitative biomarkers, minimizing reliance on self-reporting and subjective clinical judgment. Modern wearable EEG systems allow for field-based, real-time assessment, ideal for sidelines, or remote areas. EEG is non-invasive, radiation-free, and safe for frequent use, enabling longitudinal monitoring across an entire season or career. Its cost-effectiveness also makes it scalable for large populations, supporting early detection and prevention in high-risk groups. More broadly, machine learning has demonstrated adaptability across a wide spectrum of biomedical and engineering applications [20,79,80,81]. This breadth underscores the versatility of machine learning approaches for analyzing complex physiological signals and supports their continued application to EEG-based concussion classification.

### 4.3. Limitations and Future Directions

This study has several limitations. The recruited sample was smaller than the minimum estimated in a post hoc power analysis, limiting the generalizability and robustness of the findings, as well as precluding analysis by sport or symptom cluster. Although our use of cross-validation and ensemble testing strengthens the reliability of our findings, these methods cannot substitute for validation in larger cohorts. Controlling for covariates, such as hormonal birth control use which has been shown to influence functional and causal connectivity [82,83,84,85] was also not feasible due to the small sample size. Future studies should prioritize larger, diverse cohorts, longitudinal tracking across recovery stages and incorporate additional variables such as menstrual cycle, symptom profiles, and sport type to refine model specificity and interpretability. The observation that several consistently misclassified controls were rugby players also highlights the need to account for potential subconcussive exposure when refining model specificity. In addition, all data were collected at a single time point within 30 days of injury; longitudinal studies are needed to track changes in connectivity and classification performance over time.

In this study, we addressed interpretability through source-based causal connectivity analysis, which links model performance to mechanistic insights; future work will integrate additional explainable AI approaches to enhance clinical trust and transparency. From a translational standpoint, coupling high-performance classifiers with mechanistic insight can improve clinician confidence in AI-assisted diagnosis, guide targeted rehabilitation strategies, inform return-to-play/work decisions, and generate testable, network-level hypotheses about injury and recovery. In addition, the pediatric focus is critical: developing brains present unique developmental trajectories and sex-specific responses. Models should be trained, validated, and interpreted with these issues in mind.

## Figures and Tables

**Table 1:** Demographic and concussion symptom assessment data.

	Non-Concussed (*n* = 15)	Concussed (*n* = 11)
Age (years)	21 ± 1.88	20 ± 2.46
Time since injury (days)	-	10 ± 6.85
Previous diagnosed concussions to date	-	2.25 ± 1.39
SCAT5, total number of symptoms	-	14.88 ± 5.48
SCAT5, severity of symptoms	-	34.88 ± 22.53

All values are presented as mean ± standard deviation.

**Table 2:** Performance metrics of ConcNet.

Metric	Mean	95% CI
Accuracy	84.2%	81.0%/87.3%
Recall	92.9%	90.1%/95.6%
Specificity	75.5%	66.6%/84.4%
Precision	79.6%	74.4%/84.7%
F1 score	86.2%	84.4%/88.0%
AUC	0.904	0.870/0.915

**Table 3:** Top ten strongest connections ranked by the magnitude of mean |*τ*_*i*→*j*_| values for control and concussed groups.

Transmitter	Receiver	|*τ_i→j_*|	Transmitter	Receiver	|*τ_i→j_*|
RC	LC	6.54 × 10^−2^	LO	LC	7.63 × 10^−2^
LT	LC	4.83 × 10^−2^	RO	LP	7.25 × 10^−2^
LP	LC	4.61 × 10^−2^	LT	LP	7.24 × 10^−2^
RP	LC	4.58 × 10^−2^	LP	LO	7.12 × 10^−2^
RP	LP	4.58 × 10^−2^	LT	LO	7.07 × 10^−2^
LC	LT	4.44 × 10^−2^	RP	LP	6.96 × 10^−2^
RT	LP	4.24 × 10^−2^	LP	RO	6.82 × 10^−2^
RO	RT	4.10 × 10^−2^	RO	LC	6.69 × 10^−2^
RO	RP	4.05 × 10^−2^	RP	LC	6.35 × 10^−2^
RT	RP	4.01 × 10^−2^	RC	LC	6.10 × 10^−2^

**Table 4:** Statistics of the |*τ*_*i*→*j*_| distributions for the concussed and control groups.

	*M*	Median	*SD*	*COV*	Kurtosis	Skewness	*d*
Control	0.0251	0.0150	0.2920	1.1604	7.7898	1.9275	0.0502
Concussed	0.0364	0.0198	0.0444	1.2270	10.4160	2.3859	

M: mean; SD: standard deviation; COV: coefficient of variation.

## Data Availability

The data presented in this study are available on request from the corresponding author (N. Virji-Babul) due to restrictions related to the age of the participants.

## Abbreviations

The following abbreviations are used in this manuscript:

m(TBI)	Mild traumatic brain injury
RS-EEG	Resting-state electroencephalography
LSTM	Long short-term memory
CM	Confusion matrix
AU	Area under the curve
ROC	Receiver operating characteristic

## Appendix B

**Table A1:** All 90 connections ranked in the order of magnitude of group mean $\left|\tau_{\iota \to j}\right|$.

Control (*n =* 15)			Concussed (*n* = 11)		
Transmitter	Receiver	$\left|\tau_{\iota \to j}\right|$	Transmitter	Receiver	$\left|\tau_{\iota \to j}\right|$
RC	LC	6.54 × 10^−2^	LO	LC	7.63 × 10^−2^
LT	LC	4.83 × 10^−2^	RO	LP	7.25 × 10^−2^
LP	LC	4.61 × 10^−2^	LT	LP	7.24 × 10^−2^
RP	LC	4.58 × 10^−2^	LP	LO	7.12 × 10^−2^
RP	LP	4.58 × 10^−2^	LT	LO	7.07 × 10^−2^
LC	LT	4.44 × 10^−2^	RP	LP	6.96 × 10^−2^
RT	LP	4.24 × 10^−2^	LP	RO	6.82 × 10^−2^
RO	RT	4.10 × 10^−2^	RO	LC	6.69 × 10^−2^
RO	RP	4.05 × 10^−2^	RP	LC	6.35 × 10^−2^
RT	RP	4.01 × 10^−2^	RC	LC	6.10 × 10^−2^
RP	LO	3.84 × 10^−2^	LT	RP	6.02 × 10^−2^
LP	LO	3.81 × 10^−2^	LP	LC	5.70 × 10^−2^
LP	RP	3.58 × 10^−2^	RC	LP	5.70 × 10^−2^
RP	RO	3.47 × 10^−2^	LO	LP	5.60 × 10^−2^
LT	LP	3.45 × 10^−2^	LT	LF	5.43 × 10^−2^
LT	LO	3.43 × 10^−2^	LT	RC	5.30 × 10^−2^
RF	LF	3.42 × 10^−2^	LC	LF	5.27 × 10^−2^
RO	LO	3.37 × 10^−2^	LP	RP	5.09 × 10^−2^
RC	RT	3.34 × 10^−2^	LO	RP	4.85 × 10^−2^
LC	LP	3.28 × 10^−2^	RO	RP	4.81 × 10^−2^
LP	LT	3.27 × 10^−2^	RC	RF	4.75 × 10^−2^
RT	RO	3.25 × 10^−2^	RT	LP	4.67 × 10^−2^
LP	RT	3.19 × 10^−2^	RT	RF	4.65 × 10^−2^
LO	RO	3.15 × 10^−2^	LC	LO	4.61 × 10^−2^
LO	LP	3.12 × 10^−2^	RO	LT	4.47 × 10^−2^
RP	RT	3.08 × 10^−2^	RP	RO	4.30 × 10^−2^
RT	LC	2.97 × 10^−2^	RT	LO	4.27 × 10^−2^
LF	LT	2.95 × 10^−2^	RC	LO	4.25 × 10^−2^
LC	RC	2.88 × 10^−2^	LO	RF	4.22 × 10^−2^
RC	LP	2.75 × 10^−2^	LP	LF	4.21 × 10^−2^
RC	RO	2.65 × 10^−2^	RP	LO	4.19 × 10^−2^
RP	LT	2.64 × 10^−2^	RC	RO	4.16 × 10^−2^
RF	RT	2.63 × 10^−2^	LF	LT	4.06 × 10^−2^
LC	LF	2.62 × 10^−2^	LP	LT	3.99 × 10^−2^
LF	RF	2.60 × 10^−2^	RP	RF	3.98 × 10^−2^
LT	LF	2.59 × 10^−2^	LO	RO	3.92 × 10^−2^
LO	RT	2.55 × 10^−2^	RO	LO	3.79 × 10^−2^
LP	RO	2.53 × 10^−2^	LT	LC	3.78 × 10^−2^
RP	RC	2.49 × 10^−2^	RO	RF	3.71 × 10^−2^
LO	LF	2.44 × 10^−2^	LC	LP	3.69 × 10^−2^
RF	RO	2.43 × 10^−2^	LT	RO	3.63 × 10^−2^
RT	LO	2.43 × 10^−2^	LC	RF	3.55 × 10^−2^
RC	RF	2.40 × 10^−2^	RF	LO	3.49 × 10^−2^
LO	RP	2.39 × 10^−2^	RP	LT	3.48 × 10^−2^
LC	RP	2.37 × 10^−2^	RC	LF	3.30 × 10^−2^
RC	LT	2.32 × 10^−2^	LP	RT	3.19 × 10^−2^
LP	RC	2.27 × 10^−2^	LP	RF	3.19 × 10^−2^
RF	RC	2.26 × 10^−2^	LC	RP	3.14 × 10^−2^
LO	LC	2.23 × 10^−2^	LC	LT	3.13 × 10^−2^
LF	RT	2.22 × 10^−2^	RC	RP	3.05 × 10^−2^
LC	RO	2.19 × 10^−2^	LC	RO	3.02 × 10^−2^
LO	LT	2.18 × 10^−2^	LC	RC	3.01 × 10^−2^
RF	LT	2.15 × 10^−2^	RP	LF	2.98 × 10^−2^
RT	RC	2.14 × 10^−2^	LF	RF	2.95 × 10^−2^
LC	RT	2.12 × 10^−2^	LP	RC	2.79 × 10^−2^
LT	RO	2.10 × 10^−2^	RF	RT	2.77 × 10^−2^
RO	RC	2.01 × 10^−2^	LF	LP	2.76 × 10^−2^
LC	LO	1.96 × 10^−2^	LO	RT	2.67 × 10^−2^
RC	LF	1.93 × 10^−2^	LO	LT	2.66 × 10^−2^
RO	LT	1.89 × 10^−2^	RT	LC	2.66 × 10^−2^
RT	LF	1.88 × 10^−2^	LF	LC	2.55 × 10^−2^
RT	LT	1.82 × 10^−2^	RC	RT	2.55 × 10^−2^
LT	RF	1.80 × 10^−2^	LF	LO	2.53 × 10^−2^
LF	LO	1.75 × 10^−2^	RC	LT	2.51 × 10^−2^
RP	LF	1.69 × 10^−2^	RP	RT	2.49 × 10^−2^
RO	LC	1.69 × 10^−2^	LC	RT	2.46 × 10^−2^
LT	RC	1.69 × 10^−2^	RT	RO	2.44 × 10^−2^
LC	RF	1.65 × 10^−2^	RF	RC	2.43 × 10^−2^
LO	RF	1.64 × 10^−2^	RO	RT	2.40 × 10^−2^
LT	RT	1.64 × 10^−2^	LO	LF	2.34 × 10^−2^
RO	LP	1.61 × 10^−2^	RF	RO	2.29 × 10^−2^
RC	RP	1.56 × 10^−2^	LF	RT	2.24 × 10^−2^
RC	LO	1.55 × 10^−2^	RO	LF	2.16 × 10^−2^
RT	RF	1.53 × 10^−2^	RT	LF	2.12 × 10^−2^
RP	RF	1.50 × 10^−2^	RF	LF	2.02 × 10^−2^
RO	RF	1.50 × 10^−2^	RF	LC	1.97 × 10^−2^
RO	LF	1.50 × 10^−2^	LF	RP	1.94 × 10^−2^
LF	LC	1.48 × 10^−2^	LT	RT	1.87 × 10^−2^
LO	RC	1.45 × 10^−2^	RT	LT	1.81 × 10^−2^
LF	RO	1.38 × 10^−2^	RO	RC	1.80 × 10^−2^
LP	LF	1.37 × 10^−2^	RT	RC	1.79 × 10^−2^
LT	RP	1.37 × 10^−2^	RP	RC	1.65 × 10^−2^
RF	RP	1.34 × 10^−2^	RF	LP	1.58 × 10^−2^
LP	RF	1.33 × 10^−2^	LT	RF	1.49 × 10^−2^
RF	LC	1.30 × 10^−2^	RF	RP	1.49 × 10^−2^
RF	LO	1.28 × 10^−2^	LF	RO	1.40 × 10^−2^
LF	RC	1.24 × 10^−2^	RT	RP	1.39 × 10^−2^
LF	RP	9.69 × 10^−3^	LO	RC	1.36 × 10^−2^
LF	LP	9.67 × 10^−3^	RF	LT	1.34 × 10^−2^
RF	LP	8.89 × 10^−3^	LF	RC	9.12 × 10^−3^
